# Insights into the invasion biology of *Plasmodium vivax*

**DOI:** 10.3389/fcimb.2013.00008

**Published:** 2013-03-05

**Authors:** Surendra K. Prajapati, Om P. Singh

**Affiliations:** Department of Molecular Biology, National Institute of Malaria ResearchNew Delhi, India

*Plasmodium vivax* is the most widely distributed human malaria parasite outside sub Sahara regions of Africa causing huge morbidity and occasionally being severe and fatal (Kochar et al., 2005; Tjitra et al., 2008). Invasion of host erythrocytes is essential for development of disease and the process varies greatly among different malaria parasites. Merozoites of *P. vivax* and *P. berghei* (a rodent malaria parasite) primarily invade reticulocytes (Kitchen, 1938; Cromer et al., 2006) whereas *P. falciparum* invades both reticulocytes and mature erythrocytes (Pasvol et al., 1980; Mitchell et al., 1986).

Erythrocyte invasion by malaria parasites is a complex and multi-step process involving interaction between parasite ligands and host cell receptors. The molecules involved in host-parasite interactions of *P. falciparum* are well characterized and engage multiple invasion pathways (Hadley et al., 1987; Cowman and Crabb, 2006). Conversely, host-parasite interactions of *P. vivax* are poorly understood. Erythrocyte invasion by *P. vivax* is mediated by a single receptor expressed on the surface of erythrocytes and reticulocytes called Duffy receptor, or Duffy antigen receptor for chemokines (DARC) (Horuk et al., 1993). During the invasion process, Duffy receptor is recognized and bound by Duffy binding protein (DBP) of *P. vivax*. DBP is a 140 kD protein located within micronemes of merozoites (Fang et al., 1991; Adams et al., 1992) and it belongs to the family of Duffy binding like erythrocyte binding proteins (DBL-EBP). DBP is comprised of five regions based on conserved cysteine residues, and region II confers adherence to Duffy receptor on the erythrocyte surface. The absence of Duffy receptor (Duffy negative trait) confers resistance to *P. vivax* infection (Miller et al., 1976). The Duffy negative trait is conferred by a point mutation in the promoter region of the Duffy receptor gene, which abolishes erythroid expression of this receptor (Tournamille et al., 1995). Fixation of the Duffy negativity trait, and the absence *of P. vivax* infection in human populations of the African continent, supports the hypothesis suggesting that *P. vivax* cannot infect Duffy negative individuals (Miller et al., 1976).

In addition to DBP, other *P. vivax* proteins involved in recognition and binding of reticulocytes have been identified, however, their respective receptors on reticulocytes are not defined. These parasite proteins include reticulocyte-binding proteins (RBP) and merozoite surface protein-1 (MSP-1) (Galinski et al., 1992; Cantor et al., 2001; Rodriguez et al., 2002). The specificity in binding of reticulocytes is conferred by the RBP family. Members of the RBP family are found in malaria parasites of humans, simians, and rodents (Galinski et al., 1992; Keen et al., 1994; Rayner et al., 2000, 2004). Major functions of RBPs can be observed during the initial process of erythrocyte selection/recognition and invasion (Galinski et al., 1992). Two members (RBP-1 and RBP-2) of the RBP family have been characterized so far from *P. vivax* and are promising vaccine candidates (Galinski and Barnwell, 1996). However, genome sequences of several malaria parasites of human, primate, and rodent have revealed many putative RBP family-like genes. Comparative genetic analysis of members of the RBP family suggests that the family is highly evolved and conserved across malarial parasites where each parasite species encodes a range of 4–15 members (Aurrecoechea et al., 2009). *P. vivax* encodes 11 members of the RBP gene family that are believed to provide recognition and specificity in the reticulocyte binding process. Characterization of DNA sequence polymorphisms of a few members of the RBP gene family from worldwide field isolates (Rayner et al., 2005; Kosaisavee et al., 2012; Prajapati et al., 2012) reveals a level of genetic polymorphisms commonly observed in promising vaccine candidate genes of malarial parasites. This suggests that members of RBP are recognized by the host immune system.

Interestingly, several recent reports show infection of *P. vivax* in Duffy-negative individuals in African (Ryan et al., 2006; Mendes et al., 2011; Wurtz et al., 2011) and American continents (Cavasini et al., 2007a,b; Carvalho et al., 2012). These reports are highly intriguing considering the presumed dependence of *P. vivax* on Duffy receptor for invasion. Thus, Duffy receptor may not be the only gateway for *P. vivax* and alternative invasion mechanisms may exist (Mons, 1990). It is important to understand how, and under what circumstances, *P. vivax* infects to Duffy negative individuals.

*P. vivax* is the only human malaria parasite that invades reticulocytes. Each of the 11 proteins encoded by the RBP gene family has a cognate receptor on the surface of reticulocytes that is essential for *P. vivax* invasion. Therefore, understanding the invasion biology of *P. vivax* requires functional characterization of RBPs and their receptor on the reticulocyte surface. Unlike *P. falciparum*, the study of *P. vivax* is associated with technical and experimental difficulties that have impeded progress in understanding aspects of parasite genetics, such as population genetics. A major technical problem with *P. vivax* is the lack of an *in vitro* culture system. Other experimental constraints include the need of ample reticulocytes for long-term culture, a poor understanding of the biology of reticulocytes and their receptors, and a remarkably low parasitemia in *P. vivax-infected* individuals. Consequently, functional studies aiming to uncover various aspects of *P. vivax* biology such as invasion, reproduction, virulence, and development are poorly understood.

Despite these limitations, extensive efforts aimed at developing a continuous culture system for *P. vivax* have resulted in establishment of short-term *in vitro* invasion and maturation assays (Chotivanich et al., 2001; Udomsangpetch et al., 2007; Russell et al., 2008), in addition to a methods for cryopreservation of both *P. vivax* and cord blood reticulocytes (Borlon et al., 2012). These advances provide important tools for unraveling the unique biology of *P. vivax* invasion, such as identifying receptors for RBPs on reticulocytes using pull-down assays. This kind of study would not only uncover molecular mechanism underlying invasion biology but also open new avenues to look for alternative routes for *P. vivax* invasion.

Considering *P. vivax* can infect Duffy negative individuals, functional characterization of RBPs and their receptors on reticulocytes will provide new insights into the biology of *P. vivax* invasion. Given that *P. vivax* cannot invade mature erythrocytes, understanding the role of RBPs in invasion process is critical for developing a new generation of treatment therapies.
